# Correlation between the electrocardiogram amplitude detected by an implantable cardiac monitor and the implantation depth

**DOI:** 10.1111/anec.13102

**Published:** 2023-12-13

**Authors:** Yohei Kawatani, Takaki Hori

**Affiliations:** ^1^ Cardiovascular Surgery Kamagaya General Hospital Kamagaha‐Shi Japan

**Keywords:** amplitude, depth, ICM, ILR, implantable cardiac monitor, implantable loop recorder

## Abstract

**Introduction:**

Implantable cardiac monitors (ICMs) primarily use R‐R intervals in subcutaneous electrocardiograms (ECGs) to detect arrhythmias. Therefore, reliable detection of R‐wave amplitude by an ICM is vital. Since ICMs detect subcutaneous ECGs, the impact of the implantation depth should be assessed.

**Methods and Results:**

This study investigated the influence of ICM depth on R‐wave (ICM‐R) amplitude on an ECG generated by an ICM (JOT Dx; Abbott). Overall, 58 patients who underwent ICM implantation at Kamagaya General Hospital from May 2022 to April 2023 were retrospectively reviewed. The depth‐position was measured using ultrasound imaging after implantation. The depth of the ICM did not show any correlation with ICM‐R amplitude (*r* = −.0141, *p* = .294). However, the distance between the ICM and the heart surface showed a significant correlation with ICM‐R amplitude (*r* = −.581, *p* < .001). Body weight (*r* = −.0283, *p* = .033) and body mass index (*r* = −.0342, *p* = .009) were associated with ICM‐R amplitude. S wave in the V_1_‐lead was also associated with ICM‐R amplitude (*r* = .481, *p* < .001). After multivariate analysis, the distance between the ICM and heart surface and the S wave in V_1_ were independent determinants for the ICM‐R amplitude.

**Conclusion:**

The ICM‐R amplitude may be higher with the ICM implanted deeper.

## Graphical abstract

The distance between the ICM and the myocardium (D2) correlated with the ICM‐R amplitude regardless of device depth (D1).

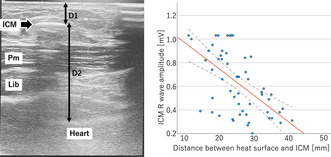

## 1 INTRODUCTION

An implantable cardiac monitor (ICM) is a device that can continuously monitor cardiac rhythm for several years. ICMs provide essential information on the relationship between symptoms and cardiac rhythms. These devices are effective in detecting syncope and atrial fibrillation, as reported in large‐sample, seminal studies, including the Randomized Assessment of Syncope Trial (Krahn et al., 2001), the Place of Reveal in the Care Pathway and Treatment of Patients with Unexplained Recurrent Syncope (Edvardsson et al., 2011), and the Cryptogenic Stroke and Underlying Atrial Fibrillation trial (Edvardsson et al., 2014). Therefore, ICMs have been recognized as a well‐established diagnostic tool for syncope and cryptogenic stroke (Brignole et al., 2018).

Implantable cardiac monitors primarily use R‐R intervals in subcutaneous electrocardiograms (ECGs) to detect arrhythmias. Therefore, reliable detection of R‐wave amplitude by an ICM is vital. Furthermore, ICMs monitor subcutaneous ECGs sensed by dipole‐electrodes placed under the skin. Consequently, the impact of the implantation depth should be assessed. However, only limited studies have investigated the correlation between the implantation depth and R‐wave amplitude.

Therefore, this study aimed to assess the association between the depth of the ICM beneath the skin and the detected R‐wave amplitudes.

## 2 METHODS

### 2.1 Study design

This retrospective study was designed to assess the ECG generated by an ICM (JOT Dx; Abbott) and investigate the influence of ICM depth. Overall, 58 patients who underwent ICM (JOT Dx; Abbott) implantation at Kamagaya General Hospital from May 2022 to April 2023 were retrospectively reviewed. The post‐implantation observation period for analysis in this study was 6 months for each patient. All patients provided written informed consent for the data analysis. The study was conducted in accordance with the Declaration of Helsinki and the applicable local legislation, and the protocol was approved by the Institutional Review Board (approval number: TGE01828‐064).

### 2.2 Data collection and definitions

Age was defined as the patient's age during the ICM implantation. Body mass index (BMI) was calculated by dividing the patient's weight (kg) by the square of their height (m). Body surface area was calculated using the following formula: weight^0.425^ × height^0.725^ × 0.007184. The clinical indications for ICM implantation included recurrent unexplained syncope and cryptogenic stroke.

**FIGURE 1:**
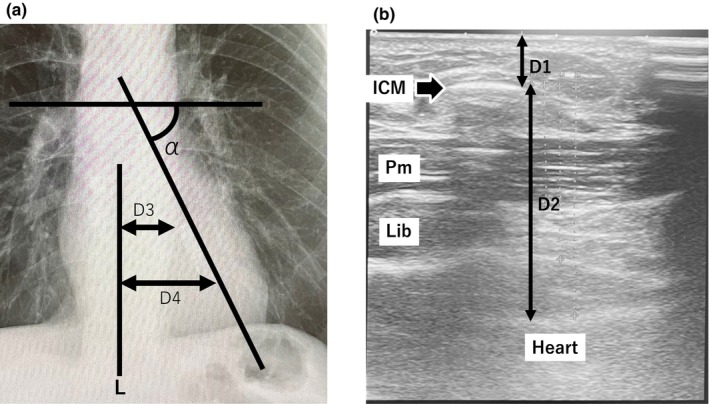
Images for measuring the position of the implantable cardiac monitor (ICM). (a) Chest radiograph. The proximal (D3) and lateral (D4) distances were the distances from the median line (L) and proximal and distal margins of the ICM. The angle was measured along the electrical axis of 12 leads (α). (b) Ultrasound image measuring the depth of the ICM. (D1); distance between the body surface and the ICM (D2); distance between the ICM and the heart surface. ICM, implantable cardiac monitor; Pm, pectoralis major muscle.

The angle and position of the ICM were manually measured on a chest radiograph, taken in a posteroanterior standing position, within 1 month of implantation. The ICM angle was defined as the angle between the horizontal and the longitudinal lines of the device (Figure 1a). Twelve‐lead ECG was performed within 1 month of implantation and the following measurements were taken: the QRS vector and amplitudes of leads II and V_1_; the S‐wave amplitude of V_1_; and the R‐wave amplitude of V_5_. The difference between the QRS vector and the ICM angle was defined as the acute angle between the ICM angle and the QRS vector.

The ICM (JOT; Abbott) uses the highest Q‐, R‐, or S‐wave amplitude sensed by the ICM as the “R‐wave” for its automatic detection of arrhythmias. The amplitude of the R‐wave detected by the ICM (ICM‐R) was defined as the R‐wave amplitude measured automatically by the ICM and interrogation program immediately after ICM implantation in a supine position. Undersensing or oversensing by the ICM‐R were both defined as the difference between the automatic detection of the ICM‐R wave by the ICM algorithm and the physician's interpretation of the ICM‐R as recorded by the ICMs.

The depth‐position of the ICM was measured using an ultrasound image obtained at the post‐operative outpatient follow‐up visits from 1 week to 1 month after implantation. The distances between the body surface and the ICM, and the ICM and the heart surface were measured (Figure 1b).

### 2.3 ICM implantation technique

Real‐time ultrasound‐guided implantation under tumescent local anesthesia (TLA) is used in our facility to achieve improved analgesia, safer procedures, and deeper implantation. TLA is an anesthesia technique for inducing sufficiently broad and deep tissue analgesia using highly diluted lidocaine (Klein, 1990). In this study, the TLA solution consisted of 100, 10, and 10 mL of saline, sodium bicarbonate, and 1% lidocaine, respectively.

**FIGURE 2:**
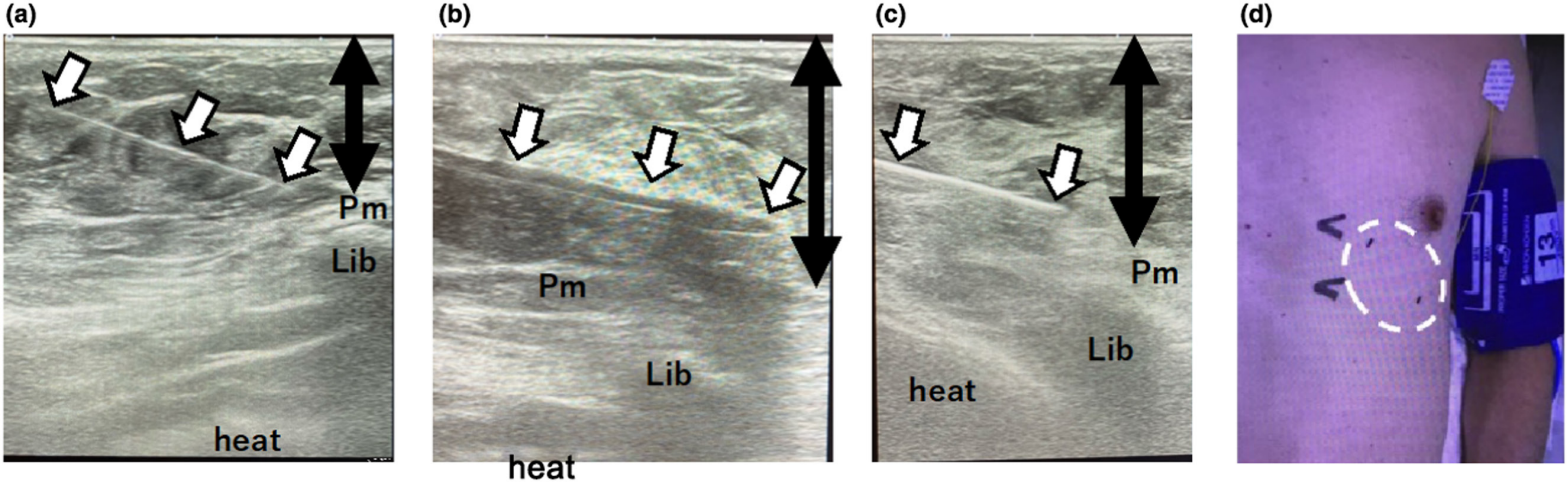
Real‐time ultrasound images during the procedures. (a) A 7 cm‐long 23‐gauge needle (arrow) is inserted into the subcutaneous tissue under real‐time ultrasound guidance. The target layer for injecting the tumescent local anesthesia (TLA) solution is denoted by the black arrow. (b) A plunger stick included in the implantable cardiac monitor (ICM) kit (JOT Dx; Abbott) (arrow) is inserted as deeply as possible into the subcutaneous tissue. (c) The ICM (arrow) is implanted. (d) Picture indicating the area of TLA application (dotted circle). Pm, pectoralis major muscle.

The TLA solution was injected using a 23‐gauge 7‐cm‐long needle under real‐time ultrasound imaging guidance (Figure 2a). Next, the TLA solution was injected into the skin and subcutaneous fat between the surface of the pectoralis major and the dermis on the ultrasound image (Figure 2a–c). TLA was induced over the ICM implantation area on the left precordium, which was approximately 10 cm long and 5 cm wide (Figure 2d). After adequate injection of the TLA solution (approximately 50–100 mL, depending on the patient's subcutaneous fat depth), an incision was made on the sternal border of the 4th left intercostal space. Subsequently, after the plunger stick for ICM implantation was inserted under ultrasound guidance (Figure 2b), the ICMs were inserted (Figure 2c). Finally, the ICM‐R amplitude was confirmed, and the wound was closed using an absorbable suture.

### 2.4 Statistical analysis

Continuous and categorical variables are presented as the median (range) and frequencies (percentages), respectively. Mann–Whitney and *χ* ^2^ tests were used to compare the continuous and categorical variables between the two groups, respectively. The correlation between ICM‐R amplitude and each continuous predictor variable was assessed using univariate linear regression. Valuables with a *p*‐value of <.05 were included in a forward stepwise multivariate model to determine the factors independently associated with ICM‐R amplitude. Among the patients' characteristics, which use similar variables for calculation, one value with the greatest correlation coefficient was included in the multivariate model to avoid collinearity. Regarding 12‐lead ECG QRS amplitudes, one value with the highest correlation coefficient among all ECG leads (12‐lead and Holter) were included in the multivariate model to avoid collinearity. Statistical analyses were performed using SPSS version 29 (IBM Corp.).

## 3 RESULTS

**FIGURE 3:**
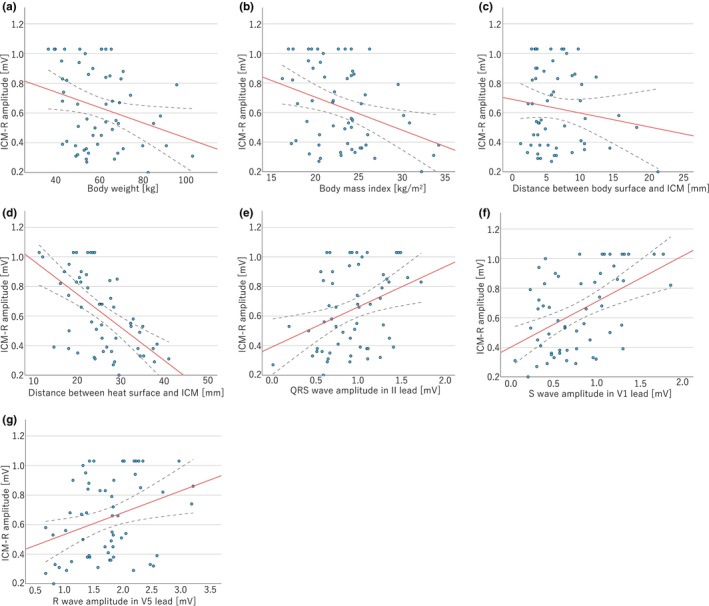
Linear plots showing R‐wave amplitudes sensed by the implantable cardiac monitor (ICM‐R). (a) Body weight, (b) Body mass index, (c) Distance between the ICM and body surface, (d) Distance between the heart wall and ICM, (e) QRS amplitude in lead II, (f) S‐wave amplitude in lead V_1_, and (g) R‐wave amplitude in lead V_5_. ICM‐R, R‐wave detected by the implantable cardiac monitor amplitude.

Overall, 58 patients were included in this study. Among these, 45 (84.9%) were males, and the median age was 75 years (16–87) (Table 1). All patients were of Asian ethnicity and almost all of their ICM implantation indications were unexplained syncopes. Body weight, body surface area, and BMI were significantly correlated with ICM‐R amplitude, but height and body surface area were not (Figure 3a,b). All ICMs were implanted through an incision on the sternal border of the 4th intercostal space. The angle of ICM implantation in the medio‐lateral position did not significantly correlate with ICM‐R amplitude. Furthermore, the difference between the implantation angle and 12‐lead ECG QRS vector was not significantly associated with ICM‐R amplitude (Table 1).

**TABLE 1:** Correlations between R‐wave detected by implantable cardiac monitor amplitude and patient characteristics and electrocardiogram.

Characteristics		Correlations with ICM‐R amplitude	
		*r*	*p*
Total number of patients	58		
Age, year	75 (16–87)	.42	.752
Sex			
Male	45 (57)		.887^a^
Female	13 (43%)		
Height (cm)	161 (140–182)	−.09	.505
Body weight (kg)	57.0 (36.5–96.7)	−.283	.033
Body surface area (m^2^)	1.57 (1.23–2.15)	−.241	.071
BMI, kg/m^2^	22.0 (16.2–34.2)	−.0342	.009
Indications for ICM			
Unexplained syncope	55 (94.8%)		.617^a^
Cryptogenic stroke	3 (5.2%)		
Implantation site			
Implantation angle (degree)	31.5 (3.2–70)	−.169	.203
Cranial/caudal location			
Incision site			
4th intercostal area	58 (100%)		—
Medial/lateral location			
Medial, delimiting the device (mm)	38 (14–64)	.111	.406
Lateral, delimiting the device (mm)	61 (30–104)	−.068	.622
Difference between implantation angle and QRS vector (degree)	−14 (−124 to 126)	−.169	.203
Vertical position/depth			
Distance between the body surface and ICM (mm)	5.1 (1.2–21.2)	−.141	.294
Distance between ICM and heart surface (mm)	24.2 (16.9–41.0)	−.581	<.001
Electrocardiogram			
12‐Lead QRS complex vector (degree)	47 (−63 to 176)	.049	.713
12‐Lead amplitudes (mV)			
QRS complex in Lead II	0.91 (0.20–1.57)	.355	.006
QRS complex in V_1_	1.01 (0.38–1.81)	.256	.055
S‐wave in Lead V_1_	1.01 (0.38–1.81)	.486	<.001
R‐wave in Lead V_5_	1.81 (0.800–3.19)	.328	.012
ICM			
ICM‐R amplitude (mV)	0.58 (0.29–1.03)		
Undersense of R wave	1 (1.8%)		
Oversense of R wave	0 (0%)		

Abbreviations: BMI, body mass index; ECG, electrocardiogram; ICM; implantable cardiac monitor; ICM‐R, R‐wave detected by the implantable cardiac monitor amplitude.

^a^ Compared using *χ* ^2^ test.

The depth of ICM implantation did not show any correlation with ICM‐R amplitude (Figure 3c). However, the distance between the ICM and the heart surface was significantly correlated with ICM‐R amplitude (Figure 3d).

QRS wave amplitudes in lead II, QRS wave amplitude in lead V_1_, S‐wave amplitude in lead V_1_, and R‐wave amplitude in lead V_5_ were correlated with the ICM‐R wave amplitude, with SV_1_ showing the highest collinearity (*r* = −.486, *p* < .001) (Figure 3e–h).

After a forward stepwise multivariate linear regression analysis, the distance between the ICM and heart surface and that of the 12‐lead ECG S‐wave amplitude in V_1_ were the independent determinants of ICM‐R amplitude (Table 2).

**TABLE 2:** Linear regression analysis for determinants of R‐wave amplitude sensed by the implantable cardiac monitor (ICM‐R).

Variables	*β*	Standard error	95% CI		*p*‐Value
Body mass index	.037	.008	−.014	.019	.777
Distance between ICM and heart surface	−.47	.005	−.028	−.008	<.001
S‐wave in lead V_1_	.339	.076	.064	.368	.006

Abbreviation: CI, confidence interval.

## 4 DISCUSSION

### 4.1 Determinants of ICM‐R amplitude

The current ICM algorithms are mainly based on the R‐R interval (Assaf et al., 2022). Therefore, the ICM‐R amplitude is essential for diagnostic procedures using ICMs and higher ICM‐R amplitudes should be obtained for accurate diagnosis (Brignole et al., 2008; Kida et al., 2022). Furthermore, the implantation depth has been assumed to be associated with ICM‐R amplitude since ICMs detect subcutaneous ECG. This study was conducted to clarify this hypothesis.

In this study, the distance between the heart surface and the ICM showed a significant correlation coefficient. Multivariate linear regression analysis also revealed that the distance between the ICM and the heart surface was an independent determinant for ICM‐R amplitude. However, the distance between the body surface and the ICM, i.e., the ICM depth, did not correlate with ICM‐R amplitude. According to these results, we propose that the higher the ICM‐R amplitude, the closer to the heart the ICM is, regardless of its depth.

There are principles that increase the distance between the myocardium and the recording electrodes, such as a thick chest wall or various intrathoracic conditions. These lower the electrical signal reaching the electrode and decrease the detected amplitude (Galen, 2008). For example, the efficient conducting properties of fluids reduce the sensed amplitude by causing short circuits. Conversely, aerated lung tissue and adipose tissue reduce the amplitude because of their lower conductivity (John & Michael, 2021). This study revealed that the ICM‐R amplitude follows these physiological principles. With fewer tissues between the myocardium and the ICM electrode, the distance becomes shorter and the ICM‐R amplitude increases.

Few reports have analyzed the impact of the distance between the ICM and the heart. A study reported that manual elevation of the breast lowers the QRS amplitude sensed by the ICM in patients whose ICM had been implanted shallowly in the breast (Perrin et al., 2018). In this situation, the distance between the ICM and the heart is larger, lowering the ICM‐R amplitude. Thus, this report strengthens our conclusion.

Obesity is reportedly associated with low QRS amplitude in ICMs (Bisignani et al., 2023; Chrysostomakis et al., 2003; Forleo et al., 2021). In the univariate linear regression analysis, our study revealed that BMI was associated with ICM‐R amplitude; however, BMI was not an independent determinant of ICM‐R amplitude after multivariate regression. Therefore, we hypothesized that the BMI value includes confounding factors for determining the ICM‐R amplitude. The confounding factors are assumed to be the thickness of the tissue between the ICM and the heart, which directly affect the ICM‐R amplitude. Bisignani et al. (2023) also described a similar hypothesis.

Ultimately, we propose that we should implant ICMs as deep as possible in the subcutaneous fat to increase proximity of the ICM to the myocardium regardless of the depth beneath the skin. Reports have described that the female sex (Ahn et al., 2021; Forleo et al., 2021) and increased height (Ahn et al., 2021) are associated with a lower QRS amplitude sensed by ICMs. However, in our study, these characteristics did not correlate with ICM‐R amplitude. We assume that these categories include many confounding factors, and that body‐build cannot be simply categorized. Therefore, these results may differ depending on the characteristics of the patient cohort among studies. Furthermore, we hypothesize that the distance between the ICM and the heart may be one of the confounding factors.

Several studies have analyzed the association between 12‐lead ECG examination and QRS amplitudes sensed by ICMs. QRS amplitudes in leads V_1_, _2_, _4_, _5_, _6_ (Brignole et al., 2018) and in aVR (Ahn et al., 2021) have been reported to be correlated with QRS amplitude in ICMs. However, in our study, S‐wave amplitude in V_1_ correlated with ICM‐R amplitude. Additionally, S‐wave amplitude in V_1_ was an independent determinant after multivariate regression analysis. As described in the methods section, the automatic integration program and algorithm for detecting arrythmias in an ICM uses ICM‐R wave, the tallest wave among the Q‐, R‐, and S‐waves. ICM‐R is not the voltage between the positive and negative amplitudes of the QRS complex previously studied. Therefore, we propose that in the case of pre‐mapping for surface ECG, S‐wave amplitude in V_1_ can be used rather than QRS amplitude.

Furthermore, subcutaneous ECG amplitude is similar to the proximal portion of body surface bipolar ECG (van Dam & van Oosterom, 2007). Although precordial leads in 12‐lead ECG are not bipolar ECG, the active pole is on the precordium. Naturally, the amplitude sensed by V_1_ lead ECGs is associated with the ICM‐R amplitude, because the V_1_ electrode is the nearest precordial lead to the proximal electrode in the ICM.

Pre‐mapping of body surface bipolar ECG was previously performed to obtain better ICM‐R amplitude (Zellerhoff et al., 2000). However, current manufacturers do not recommend pre‐mapping the position of ICMs. In the user manual, the recommended implantation position is at the sternal border of the 4th intercostal space, at a 0–90° angle determined by the implanter (Abbott, 2023), with no requirement for pre‐mapping. Notably, the technique obtained a sufficient ECG on ICMs (Piorkowski et al., 2019). In this study, we used ultrasound images to determine the angle required to ensure that the ICM was implanted directly above the heart. We believed this method enabled us to achieve an “implant alongside heart” so that the ICM could be closer to the myocardium. Ahn et al. reported that a horizontal angle gained the highest ICM‐R amplitude (Ahn et al., 2021), whereas other reports describe that implantation angle was not associated with lower ICM‐R amplitude or undersensing of the ECG (Kida et al., 2022; Korada et al., 2020). Furthermore, the implantation angle and 12‐lead ECG vector similarity is associated with higher ICM‐R amplitude (van Dam et al., 2009), although a report showed that the value was not associated with ICM‐R amplitude (Ahn et al., 2021).

Moreover, the implantation angle, similarity of the implantation angle, and QRS vector medial‐lateral position were not correlated with the ICM‐R amplitude in our study.

According to these controversial findings, ICM‐R amplitude is believed to be affected by multiple factors, such as the anatomical location of the heart, electrophysiological characteristics of the myocardium, underlying pathologies of the heart, three‐dimensional condition of the ICM and the myocardium, and patient body‐build, among others. Therefore, the implantation angles or locations are not single determinants of the ICM‐R amplitude.

### 4.2 Anesthesia and implantation technique

Under conventional local anesthesia, the anesthetized area is narrow, and the depth is limited to the skin and superficial subcutaneous tissue. Additionally, without real‐time imaging beneath the skin, ICMs cannot be deeply implanted into the subcutaneous fat. Therefore, we used real‐time ultrasound‐guidance for TLA anesthesia and implantation.

Tumescent local anesthesia is a modified local anesthesia technique used to achieve sufficient analgesia among broad and deep tissue, which was originally introduced for liposuction procedures (Bush & Hammond, 1999). In the original TLA technique, the TLA solution comprises lidocaine, epinephrine, and bicarbonate diluted with saline. However, in this study, we used a TLA solution without epinephrine (1% lidocaine, 10 mL; sodium bicarbonate, 10 mL; and normal saline, 100 mL) because the patients who received ICMs had suspected arrhythmias. Because the solution contained highly diluted lidocaine, we could inject a large amount of the solution, enabling sufficient anesthesia of the broad and deep subcutaneous tissue and skin.

Recently, TLA has been applied in many skin and subcutaneous tissue surgeries, including saphenous vein insufficiency (Kawatani et al., 2019). Additionally, TLA is currently performed under real‐time ultrasound guidance as a safe technique to induce accurate depth.

Tumescent local anesthesia is also used in cardiac implantable electronic device surgeries. We have previously described the application of TLA for pacemaker implantation (Kawatani & Hori, 2022), and Romero et al. applied the technique for subcutaneous defibrillator implantation (Romero et al., 2021). This technique can provide a deeper, safer, and more accurate ICM implantation.

### 4.3 Limitations

This study had some limitations. First, this was a single‐center study with a small number of patients and without a control group. Second, only one ICM device (JOT; Abbott) was included. Each device has different specifications for sensing subcutaneous ECG, (Bisignani et al., 2018) including variable electrode placements and algorithms. Furthermore, R‐wave amplitude sensed by other devices cannot be easily compared. Third, all patients were Asian. Therefore, studies with larger sample sizes and multiple centers and ICM devices are needed to improve generalizability.

There were no patients who experienced complication related to ICMs during observation period. In addition, only one patient experienced undersense of ICM‐R by ICM automated algorithm. The results demonstrated that the technique's capability in clinical implications.

## 5 CONCLUSION

The distance between the ICM and the myocardium correlated with the ICM‐R amplitude regardless of device depth. ICMs should be implanted as deeply as possible in the subcutaneous tissue to ensure good proximity to the heart. Real‐time ultrasound‐guided TLA and implantation techniques are useful to achieve precise deeper placement.

## AUTHOR CONTRIBUTIONS

YK and TH contributed equally.

## FUNDING INFORMATION

This study is self‐funded.

## CONFLICT OF INTEREST STATEMENT

There is no conflict of interest to declare.

## ETHICS STATEMENT

This study was approved by the local ethics committee.

## PATIENT CONSENT STATEMENT

All patients provided written consent for participation in and publication of this study.

## PERMISSION TO REPRODUCE MATERIAL FROM OTHER SOURCES

Not applicable.

## CLINICAL TRIAL REGISTRATION

None.

## Data Availability

All data supporting the manuscript is shown in the manuscript.
